# The influence of duodenally-delivered Shakuyakukanzoto (*Shao Yao Gan Cao Tang*) on duodenal peristalsis during endoscopic retrograde cholangiopancreatography: a randomised controlled trial

**DOI:** 10.1186/s13020-016-0125-6

**Published:** 2017-01-09

**Authors:** Haruka Fujinami, Shinya Kajiura, Jun Nishikawa, Takayuki Ando, Toshiro Sugiyama

**Affiliations:** 1 Department of Endoscopy, Toyama University Hospital, Toyama, Japan; 2 Department of Gastroenterology, Graduate School of Medicine and Pharmaceutical Science, University of Toyama, Sugitani 2630, Toyama City, Toyama 930-0194 Japan

## Abstract

**Background:**

Anti-cholinergic agents may be used to inhibit duodenal peristalsis, but they may have adverse effects. Shakuyakukanzoto (*Shao Yao Gan Cao Tang*) has an anti-spasmodic effect and has been used before for oesophagogastroduodenoscopy and colonoscopy. This randomised clinical trial aimed to evaluate the inhibitory effect of Shakuyakukanzoto on duodenal peristalsis, and its usefulness when administered into the duodenum just before endoscopic retrograde cholangiopancreatography (ERCP).

**Methods:**

Participants were recruited between June 2008 and December 2010. All were aged ≥18 years and provided written informed consent. Exclusion criteria were: acute pancreatitis, a history of ischemic heart disease, prostatic hypertrophy or glaucoma, and altered/postsurgical upper gastrointestinal anatomy. The recruited participants were randomly assigned to the Shakuyakukanzoto group and control group. Shakuyakukanzoto 100 mg/mL solution or placebo (warm water) was administered directly as a spray into the duodenum during endoscopy. Efficacy was evaluated by observing the extent of duodenal peristalsis and assessing the difficulty of cannulating the common bile duct, the required time (RT) from administration to inhibition of duodenal peristalsis and the stop duration time (DT, the duration for which peristalsis was inhibited). Side effects were evaluated by measuring serum potassium concentration after ERCP.

**Results:**

Of 28 participants, 15 were assigned to the Shakuyakukanzoto group and 13 to the control group. Duodenal peristalsis was inhibited in eight of the 10 eligible participants (80.0%) in the Shakuyakukanzoto group and none (0%) of the nine eligible participants in the control group (*P* = 0.026). In the Shakuyakukanzoto group, mean RT (±standard deviation) was 76.0 ± 23.9 s and DT was 11.3 ± 4.2 min. No adverse effects were observed in the Shakuyakukanzoto group during or after ERCP.

**Conclusion:**

Duodenal peristalsis can be inhibited by spraying Shakuyakukanzoto solution directly into the duodenum.

*Trial registration* UMIN Clinical Trials Registry (UMIN-CTR) UMIN000011469

**Electronic supplementary material:**

The online version of this article (doi:10.1186/s13020-016-0125-6) contains supplementary material, which is available to authorized users.

## Background

Endoscopic retrograde cholangiopancreatography (ERCP) has become increasingly important in the diagnosis and treatment of pancreatic and biliary diseases [1]. It is important to obtain a clear view, without duodenal peristalsis, to perform ERCP safely and effectively. Anti-spasmodics such as hyoscine-*N*-butylbromide or glucagon are often used to inhibit duodenal spasm [2], but their systemic use may cause adverse events, including dry mouth, urinary retention, orthostatic hypotension, palpitations, hyperglycaemia and anaphylaxis. Furthermore, these drugs are contraindicated in participants with ischaemic heart disease, prostatic hypertrophy, glaucoma and diabetes mellitus [3, 4].

Shakuyakukanzoto (*Shao Yao Gan Cao Tang*), an aqueous mixture of extracts of *Paeoniae radix* (*Shakuyaku, Shao Yao*) and *Glycyrrhizae radix* (*Kanzo, Gan Cao*), is reported to rapidly reduce abdominal pain and muscular cramps [5, 6], and suppress contraction of the ileum [7]. We have previously reported the inhibitory effect of Shakuyakukanzoto on duodenal peristalsis during ERCP [8], a finding that was later corroborated by Sakai et al. [9]. This randomised clinical trial aimed to evaluate the inhibitory effect of Shakuyakukanzoto on duodenal peristalsis and its utility when administered directly into the duodenum just before ERCP.

## Methods

### Study design

**Fig. 1:**
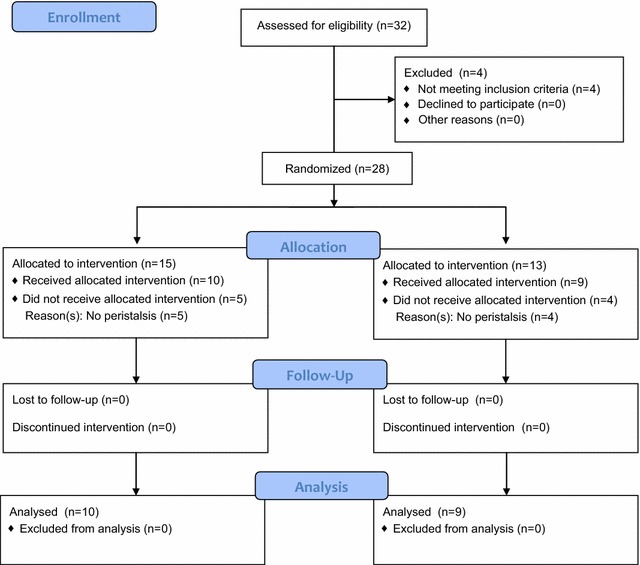
CONSORT flow diagram of enrolled and randomised participants. Recruitment, group allocation and retention of study participants

This was a prospective, randomised, placebo-controlled trial to investigate the effectiveness of Shakuyakukanzoto solution on intestinal peristalsis. A CONSORT flow diagram of the study protocol is presented in Fig. 1 [10]. The study was approved by the Ethics Committee of the University of Toyama, Toyama, Japan (Additional file 1), and informed consent was obtained from all participants (Additional file 2). Participants scheduled for ERCP at Toyama University Hospital were invited at this study between June 2008 and December 2010. Inclusion criteria were: (1) participants ≥18 years old, with (2) the capacity to provide written informed consent. Exclusion criteria were: (1) acute active pancreatitis; (2) a history of ischemic heart disease, prostatic hypertrophy or glaucoma, and (3) altered or postsurgical upper gastrointestinal anatomy. Randomization was achieved by a computer-generated list of numbers to assign group allocation.

### ERCP

Endoscopic retrograde cholangiopancreatography was performed by one of four trained endoscopists, each with at least 5 years of experience (HF, SK, JN and TA). Images of all procedures were recorded digitally. All participants were administered midazolam 5 mg (Astellas Pharma Inc., Tokyo, Japan) intravenously before the procedure, and heart rate and peripheral oxygen saturation were monitored by pulse oximetry during the procedure. We prepared a 100 mg/mL Shakuyakukanzoto solution by dissolving 5.0 g Shakuyakukanzoto extract (TJ-68; Tsumura Co., Tokyo, Japan) in 50 mL of warm water, while 50 mL of warm water was used as the placebo control. Both solutions were administered at 36 °C by spraying directly towards the major papilla of the duodenum through the endoscope. Those cases with no duodenal peristalsis at the major papilla were excluded from the study, and the study drug was not administered.

### Evaluation of duodenal peristalsis

We measured duodenal peristalsis during ERCP using a four-grade scoring system of the degree of peristalsis and the difficulty of cannulation previously described by Niwa et al. [11]. The four scores used were as follows: (+0) no peristalsis of the duodenum during ERCP, cannulation was easy to perform; (+1) slight peristalsis of the duodenum, cannulation was easy to perform; (+2) moderate peristalsis of the duodenum, cannulation was difficult to perform; and (+3) severe peristalsis of the duodenum, cannulation could not be performed.

### Efficacy and side effects of Shakuyakukanzoto solution

**Fig. 2:**
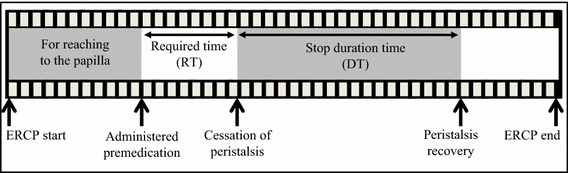
Required time and duration time of study drug during ERCP. Required time (RT): from study drug administration to cessation of peristalsis. Stop duration time (DT): from cessation to recovery of peristalsis. Both were obtained from the digital recording made of each ERCP

The primary efficacy was inhibition of duodenal peristalsis, calculated as the proportion of participants scoring either +0 or +1 after treatment. To further assess the effects of Shakuyakukanzoto solution, we reviewed the digital recordings of ERCP to measure the required time (RT) from administration of the study drug until peristalsis was diminished, and the duration time (DT) of inhibition of peristalsis (Fig. 2). As hypokalaemia has been reported as a side effect of Shakuyakukanzoto [12], we measured serum potassium concentration before and 24 h after ERCP.

### Statistical analysis

The primary outcome criterion was the efficacy rate of Shakuyakukanzoto compared with placebo. The sample size calculation for this study was based on the effective rate achieved in a previous trial, with the response rates in the Shakuyakukanzoto and control groups expected to be 70 and 10%, respectively. The no-peristalsis rate was expected to be 40% [9, 13]. The target sample size required to detect a difference in the response rate between the groups with a significance level of 5% and a power 90% was 13 per group, including a 40% dropout rate. Data are expressed as mean ± standard deviation (SD). Either Fisher’s exact test or Student’s *t* test were used to compare paired data. A *P* value less than 0.05 was considered statistically significant. All statistical analyses were performed using the Statview 5.0 (Abacus Concepts Inc., Berkeley, CA, USA).

## Results

In total, 149 ERCP procedures were performed during the study period, and 32 participants were enrolled into the study. Four were excluded due to exacerbation of acute pancreatitis and/or cholangitis. The remaining 28 participants fulfilled the inclusion criteria and were randomly allocated to one of the two groups: 15 participants to the Shakuyakukanzoto group and 13 to the placebo control group. Five participants in the Shakuyakukanzoto group and four in the control group were excluded as no duodenal peristalsis was evident at duodenoscopy. Consequently, we subjected the data of 10 and nine participants from the Shakuyakukanzoto and control groups to analysis, respectively. Participants’ demographic and clinical characteristics are summarised in Table 1. There was no significant difference in sex, mean age or indication for ERCP between the groups. Duodenal peristalsis was inhibited in eight of the 10 participants (80.0%) in the Shakuyakukanzoto group and none (0%) of the control group (*P* = 0.026; Table 2). Mean RT and DT for Shakuyakukanzoto were 76.0 ± 23.9 s and 12.4 ± 5.0 min, respectively. There was no significant difference in the serum potassium concentration in the Shakuyakukanzoto group before or after the procedure (4.1 ± 0.3 mEq/L versus 4.3 ± 0.3 mEq/L, respectively, *P* = 0.192; Table 2).

**Table 1:** Participants’ demographic and clinical characteristics

	Shakuyakukanzoto group	Control group	*P* value
Participants	10 (52.6)	9 (47.4)	
Male	7 (70.0)	6 (66.7)	1.000^a^
Age (years old)	69.1	71.2	0.708^b^
Diagnostic ERCP	7 (36.8)	3 (15.8)	0.179^a^
Therapeutic ERCP	3 (15.8)	6 (31.6)	

Data are presented as number (proportion, %) or mean ± standard deviation

^a^ Fisher’s exact test was used to assess statistical significance

^b^ Student’s *t* test was used to assess statistical significance

**Table 2:** Efficacy and safety of *Shakuyakukanzoto*

	Shakuyakukanzoto group	Control group
Ceased peristalsis	8 (80.0)	0 (0)
Required time (s)	76.0 ± 23.9	ND
Stop duration time (min)	11.3 ± 23.9	ND
Potassium concentration (mEq/L)		
Before procedure	4.1 ± 0.3	4.0 ± 0.3
24 h after procedure	4.3 ± 0.3	4.2 ± 0.2

*ND no data. The required time and stop duration time in the control group were not measured because inhibition of duodenal peristalsis was not achieved with the placebo

Data are presented as number (proportion, %) or mean ± standard deviation

## Discussion

To the best of our knowledge, this is the first placebo-controlled study that has shown that Shakuyakukanzoto is an effective and safe anti-spasmodic premedication for ERCP. In a previous observational study [9], Shakuyakukanzoto acted as an anti-spasmodic agent and abolished duodenal peristalsis in the majority of participants to whom it was administered, but this was not a randomised, controlled study and the extent of suppression of peristalsis was not measured. In this study, we excluded participants in whom duodenal peristalsis was not evident at duodenoscopy, and assessed peristalsis and its influence on the technical difficulty of cannulating the common bile duct using the scoring system previously described by Niwa et al. [11].

Gastrointestinal peristalsis may be an impediment to accurate endoscopic examination. Intramuscular or intravenous administration of an anti-cholinergic agent such as hyoscine-*N*-butyl bromide is generally required to abolish peristalsis [14]. Administration of an anti-cholinergic drug may, however, cause potentially serious complications, including cardiovascular events, urinary retention and ocular hypertension [4]. Glucagon may also be used to reduce peristalsis, but while it has fewer adverse effects on the cardiovascular system, it may induce hyperglycaemia [15]. Therefore, these drugs are not recommended for participants with cardiac disease, glaucoma, prostatic hyperplasia or diabetes mellitus [4, 15].

Shakuyakukanzoto has two active components. Paeoniflorin is a bioactive component of *Paeoniae radix*, and reportedly exhibits anti-coagulant [16], neuromuscular blocking [17–23], immunoregulating [24] and anti-hyperglycaemic effects [25]. Glycyrrhizic acid is a bioactive component of *Glycyrrhizae radix* and is reported to have anti-inflammatory [26] and hepatoprotective activity [27], and inhibit anti-platelet aggregation [28] and formation of peptic ulcers [29, 30]. These two components may exert synergistic effects. Although paeoniflorin is poorly absorbed in the gastrointestinal tract and has low bioavailability [31, 32], its absorption is significantly improved when administered orally in Shakuyakukanzoto solution [33].

Rather than administering standard anti-spasmodic drugs, or administering Shakuyakukanzoto orally, we sprayed Shakuyakukanzoto directly into the duodenum during duodenoscopy, and found that duodenal peristalsis was inhibited in 80% of cases. These effects were likely observed because the *Glycyrrhizae radix* reportedly inhibits acetylcholine-induced contraction and the contractile machinery of smooth muscle, while *Paeoniae radix* inhibits neurogenic contraction in the small bowel (the latter reportedly inhibits peristalsis in guinea pig ileum and in mouse jejunum and ileum) [7, 34]. Although paeoniflorin and glycyrrhizin may be ineffective when applied individually, they are recognised to block neuromuscular synapses when applied in combination in animal models [21].

Peppermint oil, a major constituent of which is menthol, also inhibits the contraction of smooth muscle of the gastrointestinal tract [35–37]. Instillation of peppermint oil into the colon during colonoscopy reduces spasm and reduces the need for intramuscular or intravenous anti-spasmodic agents during endoscopic examination [13, 38, 39]. The mechanism of smooth muscle relaxation brought about by peppermint oil has been investigated in models using the smooth muscle of guinea pigs and mice.

The brown colour of Shakuyakukanzoto solution might affect endoscopic examination by obscuring the entrance to the common bile duct; however, ductal cannulation was possible in all cases in which Shakuyakukanzoto was administered. Nevertheless, its distinctive colour made blinding of the study to the endoscopist impractical.

The intraluminal administration of a rapidly acting agent that directly affects smooth muscle has obvious advantages over the systemic administration of an anti-cholinergic drug. In this study, we evaluated the efficacy of Shakuyakukanzoto administered directly into the duodenum immediately before ERCP. We have also found that Shakuyakukanzoto sprayed directly onto the duodenal papilla significantly reduced serum amylase concentration 1 h and 1 day after ERCP [40]. Although we assessed only ERCP, our findings might also apply to other endoscopic examinations, such as upper endoscopy, balloon endoscopy and colonoscopy.

## Conclusion

Duodenal peristalsis can be inhibited by spraying Shakuyakukanzoto solution directly into the duodenum.

## Additional files

**Additional file 1.** Approval of ethics committee.

**Additional file 2.** Written consent form.

## Abbreviations

DT: stop duration time
ERCP: endoscopic retrograde cholangiopancreatography
RT: required time
