# Supplementary material for: The influence of duodenally-delivered Shakuyakukanzoto (Shao Yao Gan Cao Tang) on duodenal peristalsis during endoscopic retrograde cholangiopancreatography: a randomised controlled trial
Source: Chin Med. 2017 Jan 9;12:3. doi: 10.1186/s13020-016-0125-6 (PMC5223528; doi:10.1186/s13020-016-0125-6)
Supplement: Supplementary file 1 — Additional file 1. Approval of ethics committee. [file 13020_2016_125_MOESM1_ESM.pdf]

# 審 査 結 果 通 知 書

１１7号
平成１８年5月１５日

申　請　者
医学部　第三内科学
教授　杉山　敏郎　　殿

富山大学長　　西　頭　徳　三

受 付 番 号：３９３

課　題　名：内視鏡的逆行性膵胆管造影（ERCP）の前投薬に芍薬甘草湯を用いた消化管運動抑制効果の検討

主任研究者名：医学部　第三内科学　教授　杉山　敏郎

さきに申請のあった上記課題に係る実施計画を平成１８年5月１５日開催の第１回倫理審査委員会で審査し，下記のとおり判定した。

記

| | |
|---|---|
| 判定 | 非　該　当　　○承　　認　　条件付承認<br>変更の勧告　　不　承　認 |
| 理由又は勧告 | |
