# Supplementary material for: The influence of duodenally-delivered Shakuyakukanzoto (Shao Yao Gan Cao Tang) on duodenal peristalsis during endoscopic retrograde cholangiopancreatography: a randomised controlled trial
Source: Chin Med. 2017 Jan 9;12:3. doi: 10.1186/s13020-016-0125-6 (PMC5223528; doi:10.1186/s13020-016-0125-6)
Supplement: Supplementary file 2 — Additional file 2. Written consent form. [file 13020_2016_125_MOESM2_ESM.pdf]

研究参加同意書

研究責任者　杉山　敏郎　殿

「内視鏡的逆行性膵胆管造影の前投薬に芍薬甘草湯を用いた消化管運動抑制効果」を調査する研究について、担当医(　　　　　　　　　　)から研究の目的および方法など下記の項目について十分な説明を受け、その内容について理解しましたので、本研究に参加することに同意します。

なお、本研究への参加は、自分の自由意思に基づくものであることを申し添えます。

説明を受け理解した項目は、□の中にレを付けた項目です。

□　研究の目的
□　研究の方法
□　研究への参加者（被験者）にもたらされる利益及び不利益
□　研究への参加は自由意思であること
□　研究への参加に同意しない場合でも不利益を受けないこと
□　研究への参加に同意した後でも随時これを撤回できること
□　個人情報の保護に関すること
□　研究成果の公表に関すること
□　費用負担に関すること
□　その他（　　　　　　　　　　　　　　　　　　）

平成　　年　　月　　日

（本人）
署名又は記名・捺印 ________________________ 印

(代諾者)
被験者(患者)氏名 ________________________
署名又は記名・捺印 ________________________ 印
被験者(患者)との関係____________

《記載上のご注意》
１、本人が「制限能力者」（未成年者、成年被後見人、被保佐人、被補助人）の場合は、代理権者が「代諾者」欄に署名願います。
２、本人が満１５歳以上の未成年者の場合は、本人も「本人」欄に署名願います。
